# Naturally occurring compounds as pancreatic cancer therapeutics

**DOI:** 10.18632/oncotarget.26234

**Published:** 2018-10-23

**Authors:** Ines Lohse, Erin Wildermuth, Shaun P. Brothers

**Affiliations:** ^1^ Department of Psychiatry and Behavioral Sciences, Center for Therapeutic Innovation University of Miami Miller School of Medicine, University of Miami, Miami, FL, USA; ^2^ Molecular Therapeutics Shared Resource, Sylvester Comprehensive Cancer Center, University of Miami Miller School of Medicine, Miami, FL, USA

**Keywords:** pancreatic cancer, natural compounds, curcumin, resveratrol, taxane

## Abstract

Naturally occurring small molecule compounds have long been in the spotlight of pancreatic cancer research as potential therapeutics to prevent cancer progression and sensitize chemoresistant tumors. The hope is that terminal pancreatic cancer patients receiving aggressive chemotherapy can benefit from an increase in treatment efficacy without adding further toxicity by way of utilizing natural compounds. While preclinical studies on a number of natural compounds, such as resveratrol, curcumin, rapalogs and cannabinoids, show promising preclinical results, little has translated into clinical practice, though a number of other compounds hold clinical potential. Nevertheless, recent advances in compound formulation may increase the clinical utility of these compounds.

## INTRODUCTION

Despite being the 12th most common cause of cancer diagnosis in the United States, pancreatic cancer is the 2nd most common cause of cancer death with a 5-year survival of 8.2% [1]. The high mortality rate in pancreatic cancer patients is attributed to the aggressive nature of the disease and a lack of effective treatment options [2]. While surgery in combination with chemotherapy is the most effective treatment and offers the highest chances of survival, only a minority of patients (15-20%) qualify [3], with the majority of patients receiving combination chemotherapy (gemcitabine, 5-FU (fluorouracil), Abraxane and platinum drugs) or chemoradiation [2]. There is little consensus regarding specific drugs or the sequence of treatment options [2, 4] and clinical responses are low due to the high levels of chemoresistance [5].

Pancreatic cancers are characterized by a number of complex genomic alterations that differentiate pancreatic adenocarcinomas from other malignancies. Pancreatic cancer develops through a specific series of mutational events (KRAS > CDKN2A > TP53/SMAD4) that develop gradually and independently [6]. Recent studies suggested a higher impact of losses of alleles and chromotrypsis than previously anticipated [7, 8]. To date, these mutations are believe not to represent suitable targets for therapeutic intervention in pancreatic cancer patients.

Because pancreatic cancer is typically a disease of older patients, treatments are limited by the patient’s overall health and continuous aggressive treatments are often not an option in this patient population. Declining health in combination with the aggressive nature of pancreatic tumors and high levels of drug resistance limit clinical options for successful pancreatic cancer treatment and result in rapid disease progression with high mortality shortly after presentation [9].

Natural products and synthetic small molecule compounds derived from natural chemical structures have long been in the focus of the pancreatic cancer field due to reports suggesting anti-cancer efficacy in a number of different malignancies and a low toxicity profile [10, 11]. Additionally, these compounds are often well received, some even being generally regarded as safe (GRAS) and readily taken by most patients, though they as often tend to be plagued by low bioavailability. Nevertheless, several natural compounds are currently being explored for their potential in treating patients with pancreatic cancer [11–20].

## TAXANES

**Figure 1:**
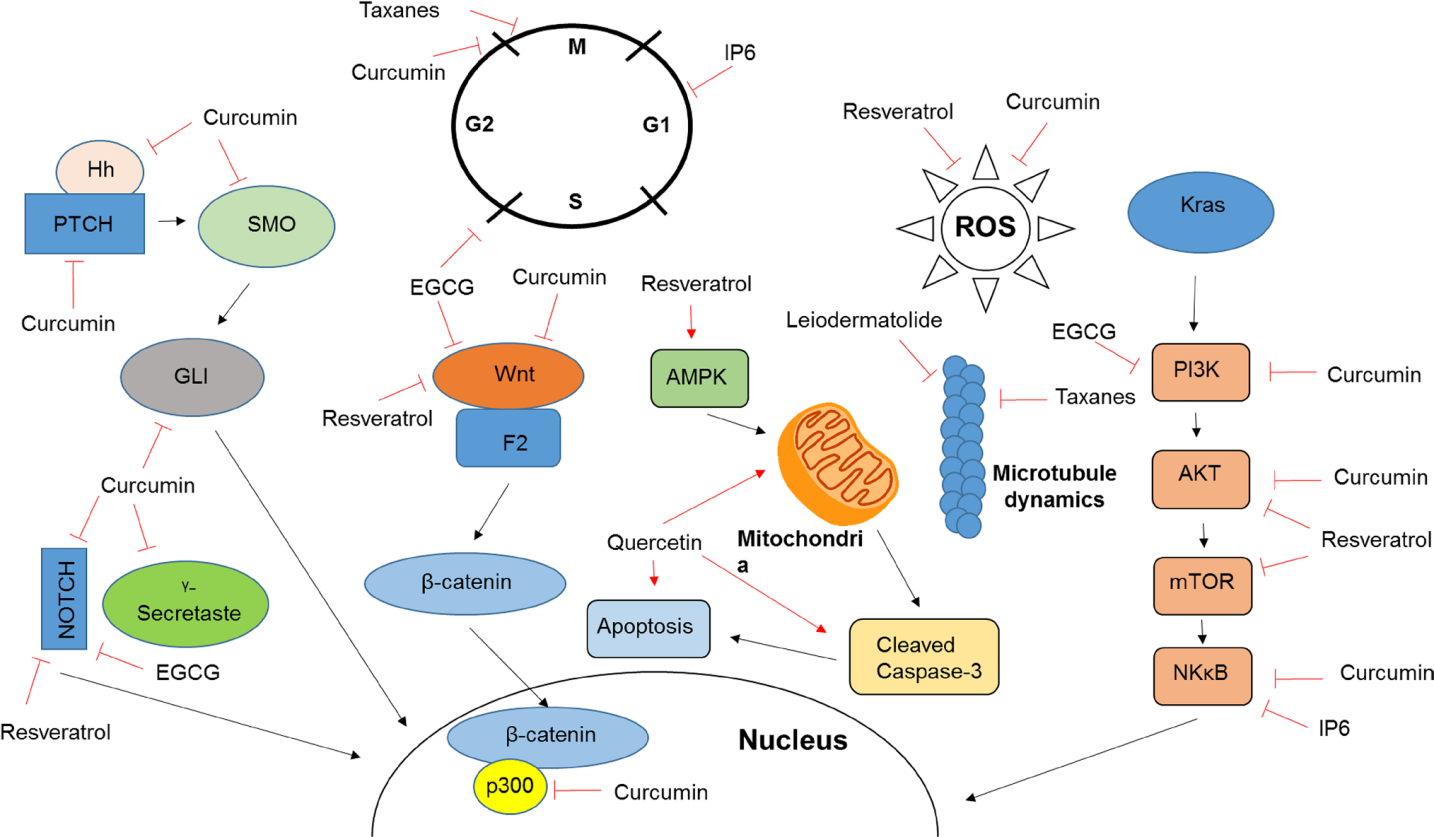
Mechanism of action of natural compounds Included are the mechanisms of action for a number of compounds discussed in this review. As further discussed in the appropriate sections, the majority of natural compounds target a wide variety of cellular pathways which contributes to the varying observations made by different studies and inhibits the transition into clinical care.

Taxanes are microtubule-stabilizing drugs that disrupt the cell cycle and are effective treatments against a range of cancers, including breast, ovarian, prostate, urothelial, and lung cancer [21–25]. The most commonly used taxanes are paclitaxel, discovered in the 1970s and derived from the western yew tree, and docetaxel, discovered in 1981 and derived via esterification of 10-deacetylbaccatin II, which can be found in the European yew tree [26]. Paclitaxel and docetaxel are hydrophobic compounds characterized by a taxane ring core, estherification at the C-13 position with a complex ester group and an unusual fourth ring at the C-4,5 position, with docetaxel differing from paclitaxel by only two moieties. These slight chemical differences result in different effects on the cell cycle. While paclitaxel inhibits the cell-cycle progression at the G2-M phase checkpoint, treatment effects of docetaxel are most prominent in S phase. Taxanes promote the assembly of microtubules and prevent their depolymerization, thus interfering with a number of normal cellular functions that depend on changes in the microtubule network. Similar to other natural compounds, taxanes have been reported to display anti-tumor effects that are not directly related to microtubule stabilization, but results from enhanced phosphorylation of Bcl-2, release of tumor necrosis factor-α (TNF-α) and a decrease in expression of TNF receptors (Figure 1) [27].

Taxanes have high activity in a wide spectrum of solid tumors (e.g. ovarian, breast, lung, head and neck, gastro-esophageal, bladder, testis, endometrium neoplasms) and are active as single agents or in combination chemotherapy. However, their clinical use is accompanied by significant side effects (neutropenia, mucositis and neuropathy).

In order to increase the tolerability of taxanes and reduce resistance, efforts have concentrated on new taxane formulations (e.g. albumin, nanoparticles, emulsions, liposomes), new taxane analogues and prodrugs. Compounds such as abraxane and docosahexenoic acid (DHA)-paclitaxel, are examples of new taxanes that have shown higher activity than paclitaxel. Both compounds display significant activity in taxane-resistant and unresponsive cancers while also exhibiting a safer toxicological profile than first-generation products.

Abraxane (nab-Paclitaxel) an albumin-paclitaxel formulation, in combination with gemcitabine was FDA-approved as a first-line treatment for pancreatic cancer based on results obtained from the MPACT phase III trial [12]. The results showed higher overall response rates (23% compared to 9%) and longer median progression-free survival rates (5.5 months compared to 3.7 months) in patients treated with the combination of nab-Paclitaxel and gemcitabine when compared to gemcitabine alone [28]. This was supported by a trial performed by Goldstein *et al.* using the combination in a large cohort of pancreatic cancer patients (*n* = 861). Patients in that study receiving the combination of gemcitabine and nab-Paclitaxel displayed increased survival (8.7 months) when compared to gemcitabine alone (6.6 months) [29]. Abraxane in combination with gemcitabine is currently a routine first-line treatment for patients with pancreatic cancer [12].

## RESVERATROL

Resveratrol is a non-flavonoid polyphenol, phytoestrogen and natural stilbene found in red wine, blueberries, cranberries and peanuts. It is known for its anti-inflammatory and antioxidant properties and has been consumed by a large part of the population in over-the-counter dietary supplements with few reports of safety issues. Studies performed in recent years also documented resveratrol as a potential anti-cancer therapeutic [30]. Resveratrol disrupts all stages of cancer development by preventing tumor initiation (antioxidant and antimutagen), reducing tumor promotion (anti-inflammatory effects and also cyclooxygenase and hydroperoxidase inhibition), inhibiting tumor growth and reducing metastatic potential (Figure 1) [30].

Resveratrol has been shown to impact a wide variety of signaling pathways (Figure 1) most of which are dependent on the microenvironmental context, such as the insulin-like growth factor system [31], Wnt signaling [32], Notch-1 signaling [33], STAT3 [33], the Akt/mTOR pathway [34] and Sirt1/AMPK [35]. Due to the many different pathways impacted and the wide range of potential interactions involved in resveratrol’s therapeutic properties, our understanding of the mechanisms at work are limited. An *in vitro* study conducted by Zou *et al.* found that resveratrol downregulated the expression of β-catenin, essential in the canonical Wnt signaling pathway [32]. Zhang *et al.* identified the Notch-1 signaling pathway as a resveratrol target in cultured vascular smooth muscle cells, with evidence of declining total and cytoplasmic levels [33]. Another study identified the STAT-1 pathway, in addition to Notch-1 and Wnt signaling, with all three signaling pathways inhibited by resveratrol in cervical cancer cells [33]. Yet another study, using T-cell leukemia cells, found that resveratrol induced apoptosis by inhibiting Akt/mTOR pathways and simultaneously upregulating p38-MAPK [34]. Still other research indicates the Sirt1/AMPK pathway as a resveratrol target [35]. Due to the many different pathways impacted and the wide range of potential interactions involved in resveratrol’s therapeutic properties, derived from ∼10,000 publications on the molecule, understanding of the precise therapeutically relevant mechanisms at work are limited [30].

Several studies have shown resveratrol to be an effective anti-cancer agent in models of pancreatic cancer [13, 36, 37] and that this effect may be mediated through leukotriene B_4_ inhibition and activation of FOXO transcription factors [38].

In a study examining the efficacy of resveratrol in combination with gemcitabine, the combination was found to inhibit, suggesting that resveratrol can improve chemotherapy outcomes, without adding to chemotoxicity [39]. Indeed, toxicity reports of resveratrol suggest that the compound is tolerated up to 1g or even 5g administered daily in humans [40]. Cell culture investigations suggest efficacy at 10 μM – 50 μM [41] and anticancer properties have been observed with concentrations as low as 5 μM [42]. A consideration in any investigation of resveratrol bioavailability is whether to measure resveratrol alone or resveratrol and its metabolites, which generally measure much higher in circulating plasma. It is possible that the benefits derived from resveratrol are as much a result from its metabolites as from the compound itself *in vivo* [42].

Resveratrol is rapidly absorbed but demonstrates a low bioavailability profile with high levels of interindividual diversity, meaning that people metabolize resveratrol differently leading to notable differences in bioavailability between individuals. The greatest bioavailability numbers indicate a *C*_max_ of approximately 4 μM when standard dosing (5 g) is used [40]. Though bioavailability can be increased by repeat dosing, a half-life of 2–5 h remains a problem for routine clinical use and rational combinations with standard of care agents [40]. With such a short half-life, even repeat dosing sees the availability of resveratrol fall more quickly over time than would be optimal for clinical use. Jupiter Orphan Therapeutics is a company working on a clinically useful formulation of resveratrol, they observe >8-fold increase in peak plasma resveratrol concentration in rats with their formulated drug compared to an equivalent unformulated reseveratrol dose (unpublished data; personal communication) creating a situation in which smaller, yet therapeutically relevant, doses can be achieved, however the clinical trials showing increased bioavailability in humans remain to be performed. This company has an open IND for phase I trials in healthy volunteers, so more bioavailability data may be available in the near future.

## CURCUMIN

Like resveratrol, curcumin has a wide range of targets and affects many cellular signaling pathways (Figure 1). Among them are the WNT/β-catenin, NOTCH, TGF/Smad, SHH, STAT3, PI3K/AKT and NF-κB/COX-2 signaling pathways, most of which play important roles in cancer development and progression [43–46]. In pancreatic cancer, curcumin induces apoptosis and inhibits cell growth and invasion *in vitro*, inhibits tumor growth and angiogenesis *in vivo* and targets cancer stem cells [17, 47–49].

Li *et al.* showed that curcumin treatment downregulates NF-κB binding and IkappaB kinase activity in pancreatic cancer cell lines. This shift was associated with a time-dependent decrease in cancer cell proliferation and increased apoptosis [50], which was further supported by Zhao *et al.* who reported that this effect is associated with an upregulation of FOXO1 expression [48]. Additionally, Ning *et al.* identified curcumin as a potential therapeutic for use against pancreatic cancer stem cells [47].

Yoshida *et al.* showed that curcumin sensitizes pancreatic cancer cells to gemcitabine in a study using gemcitabine resistant pancreatic cancer cells, and that the combination inhibits the growth of gemcitabine-resistant pancreatic cancer xenografts [51].

Few clinical trials using curcumin (Table 1), either alone or in addition to other drugs, have been conducted to date. A small trial in 21 patients who were not responding to gemcitabine alone, administered the combination of gemcitabine paired and 8 g of daily oral curcumin showed that curcumin was well tolerated and increased mean survival (161 days, with 19% of patients surviving after one year) when compared to continuation of gemcitabine alone in patients who were not responding well, who averaged 10 weeks survival rate [52]. Though studies using FOLFIRINOX in the treatment of pancreatic cancer indicate that while FOLFIRINOX displays better tumor control, the gemcitabine-curcumin combination is better tolerated [12]. However, Dhillion *et al.* showed poor bioavailability in a cohort of 25 patients, with only two patients displaying clinically relevant biological activity following the daily 8 g oral curcumin administration [53]. Though both Yoshida *et al.* and Dhillion *et al.* reported tolerance at 8 g, Epelbaum *et al.* found that 29% of patients developed abdominal pain. This study delivered mixed results, with 9% of the seventeen enrolled patients experiencing partially positive responses, 36% showing stable disease and 55% experiencing tumor reduction [54]. The development of Theracurmin, a highly bioavailable form of curcumin shown to produce a 40-fold increase in maximal blood-concentration in rats and a 27-fold increase in humans, has increased bioavailability to clinically relevant levels [55, 56] (Table 1). A phase I clinical study investigating the safety Theracurmin in cancer patients reported adverse effects associated with disease progression and not Thermacumin treatment. Though results could not confirm a corresponding decrease in NF-κB activity or cytokine levels, this study documented the safety of Theracumin [57]. A later clinical trial reported a number of adverse effects, with several patients reporting abdominal fullness and pain and showing signs of dilated colons, indicating that high bioavailability of curcumin may increase its toxicity profile. Despite a median estimated survival time of 4.4 months in the 14 clinical trial patients, three survived for more than twelve months following treatment [55, 58].

**Table 1:** Preclinical and clinical studies evaluating curcumin and theracumin

	Author	Year	Phase	Publication title
**Curcumin**	Li *et al.*	2004	Pre-clinical	Nuclear factor-κB and IκB kinase are constitutively active in human pancreatic cells, and their down-regulation by curcumin (diferuloylmethane) is associated with the suppression of proliferation and the induction of apoptosis
	Dhillon *et al.*	2008	Clinical Phase II	Phase II Trial of Curcumin in Patients with Advanced Pancreatic Cancer
	Kanai *et al.*	2011	Clinical Phase I/II	A phase I/II study of gemcitabine-based chemotherapy plus curcumin for patients with gemcitabine-resistant pancreatic cancer
	Epelbaum *et al.*	2011	Clinical Phase II	Curcumin and Gemcitabine in Patients With Advanced Pancreatic Cancer
	Bimonte *et al.*	2013	Pre-clinical	Curcumin Inhibits Tumor Growth and Angiogenesis in an Orthotopic Mouse Model of Human Pancreatic Cancer
	Ma *et al.*	2014	Pre-clinical	Curcumin inhibits cell growth and invasion through up-regulation of miR-7 in pancreatic cancer cells
	Zhao *et al.*	2015	Pre-clinical	Curcumin induces apoptosis in pancreatic cancer cells through the induction of forkhead box O1 and inhibition of the PI3K/Akt pathway
	Ning *et al.*	2016	Pre-clinical	Bulk pancreatic cancer cells can convert into cancer stem cells (CSCs) *in vitro* and 2 compounds can target these CSCs
	Zhao *et al.*	2016	Pre-clinical	Curcumin potentiates the potent antitumor activity of ACNU against glioblastoma by suppressing the PI3K/AKT and NF-κB/COX-2 signaling pathways
	Yoshida *et al.*	2017	Pre-clinical	Curcumin sensitizes pancreatic cancer cells to gemcitabine by attenuating PRC2 subunit EZH2, and the lncRNA PVT1 expression
**Theracumin**	Sasaki *et al.*	2011	Clinical Phase I	Innovative preparation of curcumin for improved oral bioavailability
	Kanai *et al.*	2013	Clinical Phase I	A phase I study investigating the safety and pharmacokinetics of highly bioavailable curcumin (Theracurmin^®^) in cancer patients
	Kanai *et al.*	2014	Clinical Phase I/II	A phase I/II study of gemcitabine-based chemotherapy plus curcumin for patients with gemcitabine-resistant pancreatic cancer

All clinical studies performed using curcumin and theracumin are summarized as an examples of clinical trials for natural compounds.

## RAPALOGS

The KRAS proto-oncogene is mutated in 90% of pancreatic cancers, leading to a constitutively active pathway resulting in rapid proliferation and increased survival [3]. KRAS mutant tumors display aberrant activation of a number of downstream signaling pathways, including phosphatidylinositol 3-kinase (PI3K) and AKT, linking KRAS mutation to activation of mammalian target of rapamycin (mTOR) [59, 60]. The mTOR pathway is a key player in many biological processes including cell growth, regulation of actin cytoskeleton, transcription, translation, cell survival and proliferation (Figure 1) [59, 61], and inhibition results in reduced protein synthesis and cell growth [61]. A number of mTOR inhibitors have been clinically investigated [14, 59]. Preclinical studies in pancreatic cancer cell lines displayed diverse effects of mTOR inhibitors on cell cycle progression, autophagy, reduced inflammation and inhibition of epithelial-to-mesenchymal transition [62–65]. However, rapid development of treatment resistance was observed in response to treatment with rapamycin through AKT phosphorylation and activation of a negative feedback loop [59]. Similar results have been observed *in vivo*, where mTOR inhibition resulted in reduced tumor growth and delayed progression in murine models of pancreatic cancer [60, 62, 66].

Clinically, rapalog monotherapy has not shown any treatment efficacy in pancreatic cancer patients, although the treatment was well tolerated [14, 67]. A study evaluating the combination of capecitabine and everolimus demonstrated a survival benefit (12.4 months) when compared to capecitabine alone (5.9 months) [68]. However, a lack of patient stratification based on biomarkers established in preclinical studies, such as a loss of or low PTEN expression and hyperphosphorylation of AKT, limits the utility of these trials for the evaluation of treatment efficacy [69, 70].

## CANNABINOIDS

Cannabis originated in Central Asia but is now grown worldwide. The cannabis plant produces a resin containing psychoactive terpenophenolic compounds called cannabinoids with the highest concentration found in the female flowers of the plant. The FDA has not approved the use of cannabis as a treatment for any medical condition and clinical trials evaluating the benefit for patients with cancer are limited.

Commercially available cannabinoids, such as dronabinol and nabilone, are approved drugs for the symptomatic treatment of cancer-related side effects. Although cannabinoids have been shown to reduce proliferation and induce apoptosis [15] in a number of tumors, including pancreatic ductal adenocarcinoma, they are mainly used as supportive therapy to reduce pain, improve sleep and improve the nutritional state of pancreatic cancer patients (Figure 1) [71].

Cannabinoids have been shown to reduce chemotherapy-induced neuropathy in animal models exposed to paclitaxel, vincristine, or cisplatin [72]. Cannabinoids reduce tumor-associated and treatment-associated pain symptoms through supraspinal, spinal, and peripheral modes of action, acting on both ascending and descending pain pathways [72, 73]. The CB1 receptor is found in both the central nervous system (CNS) and in peripheral nerve terminals where high receptor concentrations in brain regions regulating nociceptive processing [72, 73]. CB2 receptors affect mast cell receptors and keratinocytes to reduce the release of inflammatory signals and increase endogenous opioid release [72, 73].

## OTHER COMPOUNDS

Taxanes, resveratrol and curcumin are the furthest developed examples of natural compounds for the treatment of pancreatic cancer, but a number of other compounds are currently being evaluated for their therapeutic properties.

Inositol hexaphosphate (IP6) is a polyphosphorylated carbohydrate found in high-fiber foods. It has been investigated as a potential anti-cancer therapy in several different cancer types, including melanoma [73], colon cancer [72] and bladder cancer [74]. Preliminary studies have shown IP6 to be effective in decreasing pancreatic cancer cell proliferation and increasing apoptosis *in vitro* (Figure 1) [16] and described a potential therapeutic synergy between IP6 and catechin, a natural compound found in green tea [74]. *In vivo* studies on the potential benefit of IP6 to pancreatic cancer have yet to be conducted.

(-)-epigallocatechin-3-gallate (EGCG) is the most potent catechin found in green tea. *In vitro* studies have shown that EGCG inhibits cell cycle progression and induces apoptosis in pancreatic cancer cells, specifically in combination the chemotherapeutic bleomycin (Figure 1) [75]. Additionally, EGCG inhibits tumor growth, angiogenesis, and metastasis in pancreatic cancer xenografts [76].

Leiodermatolide is a polyketide macrolide found in the deep-sea sponge. It is known for its antimitotic properties and is thought to utilize a novel mechanism when compared to compounds with similar results, though the particulars of this mechanism have not yet been identified. Currently used compounds such as vinca alkaloids and taxanes induce cell cycle arrest by affecting the microtubules required for spindle formation and chromosome segregation. Preliminary data suggest that Leiodermatolide is potentially a potent inhibitor of pancreatic cancer [18].

Quercetin, a flavonoid polyphenol closely related to resveratrol that is found in many fruits, vegetables and grains, has also shown promising results *in vitro* and *in vivo*, though it has not been studied as extensively as resveratrol. Studies have shown that that quercetin sensitizes cancer cells to tumor necrosis factor-related apoptosis-inducing ligand (TRAIL) induced apoptosis, causes apoptosis *in vivo* and reduces tumor proliferation *in vivo* (Figure 1) [20, 77].

## CLINICAL IMPACT

Natural compounds play a major role as anti-proliferative agents in pancreatic cancer therapy. While taxanes have successfully transitioned into clinical use and are now part of clinical routine in pancreatic cancer treatment, this class of compounds remains one of the few examples to achieve this transition. Despite promising preclinical results using a number of natural compounds, little has translated into the clinical routine of pancreatic cancer treatment although a large number of clinical trials have been performed on various compounds. This can be attributed to unspecified mechanisms of action, low bioavailability and difficulty ensuring patients’ compliance with the dosing regimen. This is further emphasized on the examples of curcumin and theracumin (Table 1), where clinical trials performed at different centers describe vastly different study outcomes. Similar results can be observed for the majority of clinical trial involving natural compounds. While different dosing regimens and daily doses are partially responsible for these differences, large numbers of target genes (Figure 1) and yet to be elucidated mechanisms of action further complicate the development of consistent clinical trial protocols. Nutraceuticals, specifically resveratrol and curcumin are highly accessible to study participants as over-the-counter dietary supplements. Patients can consume large amounts of these compounds outside of the prescribed dosing regimen, which is particularly problematic for the analysis randomized clinical trial aiming to evaluate treatment effects and toxicity in combination with standard-of-care.

For natural compounds to become clinically relevant to pancreatic cancer treatment, these pitfalls need to be addressed. Taxanes are the only natural compound currently approved for clinical use in pancreatic cancer, though resveratrol and curcumin may be suggested as supplemental supportive care or taken by patents on their own accord.

With continuous advances in medicinal chemistry and drug formulation, will enable the improvement of natural compound-based anti-cancer drugs and facilitate a transition of these compounds into the clinic.

## CONCLUSIONS

The wide variety of mechanisms of action associated with natural compounds is problematic in terms of isolating and confirming specific cellular targets and their impact on tumor cell survival. Though recent studies identified some mechanisms of action, we are far from understanding the full spectrum of effects that natural therapeutics have on normal and cancer cells. With recent drug development efforts aiming to increase the bioavailability of natural compounds, such as resveratrol and curcumin, the clinical use of these compounds become feasible by allowing the development of rational combinations with established chemotherapeutic agents.

The combination of natural products and standard of care chemotherapy has the potential to increase quality of life and lifespan in pancreatic cancer patients, even though a number of hurdles need to be overcome for routine clinical use.
